# Prevalence and determinants of silent myocardial ischemia in patients with type 2 diabetes in Cameroon: a cross-sectional study

**DOI:** 10.11604/pamj.2022.42.41.34941

**Published:** 2022-05-16

**Authors:** Liliane Mfeukeu-Kuate, Vanessa Abongteh Meyanui, Ahmadou Musa Jingi, Valerie Ndobo-Koe, Franck Mballa, Marie Ntep- Gweth, Martine Etoa Etoga, Jean Jacques Noubiap, Eugène Sobngwi, Alain Menanga

**Affiliations:** 1 Faculty of Medicine and Biomedical Sciences, University of Yaoundé I, Yaoundé, Cameroon,; 2 Cardiology Unit, Central Hospital of Yaoundé, Yaoundé, Cameroon,; 3 Faculty of Health Sciences, University of Bamenda, Bamenda, Cameroon,; 4 Endocrinology and Diabetology Unit, Central Hospital of Yaoundé, Yaoundé, Cameroon,; 5 Centre for Heart Rhythm Disorders, the University of Adelaide, Adelaide, Australia

**Keywords:** Myocardial ischemia, type 2 diabetes, exercise stress ECG, Africa

## Abstract

**Introduction:**

diabetes mellitus is a major health problem worldwide. It is associated with high cardiovascular morbi-mortality especially coronary artery disease. Myocardial ischemia is often asymptomatic (silent myocardial ischemia) in people with type 2 diabetes mellitus thus making early diagnosis difficult. Silent myocardial ischemia is defined as the objective evidence of myocardial ischemia in the absence of chest discomfort or other angina equivalents. This study aimed to determine the prevalence and determinants of silent myocardial ischemia in a population of people with type 2 diabetes using exercise stress electrocardiography.

**Methods:**

we carried out a cross-sectional study between January and April 2019 at the National Obesity Centre of the Yaounde Central Hospital. Patients with type 2 diabetes underwent a complete clinical evaluation, blood test, resting electrocardiogram (ECG), and exercise stress ECG according to the Bruce protocol. A positive stress test was defined as horizontal or down-sloping ST depression ≥ 1mm or upsloping ST depression of 2 mm or more 0.06 to 0.08 seconds after the J point.

**Results:**

a total of 112 patients with diabetes (63 males and 49 females) were screened. The median age was 58 (IQR: 51 - 64) years. The median time from diabetes diagnosis was 8 (IQR: 5 - 12) years. Fifty-seven (50.4%) had hypertension, 78 (69.0%) had dyslipidemia, 66 (58.4%) were obese, 70 (61.9%) had poor glycemic control, and 23 (20.2%) were smokers. Sixty-five (58%) patients had a positive exercise stress ECG test. Factors independently associated with a positive stress test were abdominal obesity (aOR: 4.2, [95% CI: 1.4 - 12.8]) and Female sex (aOR: 2.5, [95% CI: 1.1 - 5.7]).

**Conclusion:**

the prevalence of silent myocardial ischemia was high in a population of asymptomatic patients. This was independently associated with abdominal obesity and female sex.

## Introduction

Diabetes mellitus is a global health problem with the greatest burden in low and middle-income settings (LMICs). The International Diabetes Federation (IDF) estimated in 2021 that about 537 million adults aged 20-79 had diabetes and predicted that in 2045, 783 million people will have diabetes, with Africa expected to have by far the highest increase in prevalence (+ 134%). Over one in four adults with diabetes are undiagnosed (about 212 million people) [1-3]. Chronic complications of diabetes lead to long-term damage, dysfunction, and failure of various organs [4]. Diabetes is a major cardiovascular risk factor. People with diabetes are three to four-fold at higher risk of cardiovascular disease than the general population [5]. Cardiovascular disease is the leading cause of morbidity and mortality in people with diabetes [6]. However, because of autonomic neuropathy, coronary artery disease (CAD) is often asymptomatic and thus underdiagnosed in people with diabetes [7].

Cardiovascular autonomic neuropathy affects both the sympathetic and parasympathetic nerves [8-10]. Damage to the sensory neurons leads to impaired signal transmission and poor or no perception of nociceptive stimuli [11]. Hence, chest pain in response to myocardial ischemia may be blunted [12]. This leads to late diagnosis, late initiation of therapy, and a more advanced stage of CAD at the time of diagnosis [13]. Sometimes, acute coronary artery events might be its initial presentation [14]. Because of the silent nature of myocardial ischemia in people with diabetes, early diagnosis through screening is therefore important [15]. Screening aims to identify patients whose prognosis could be improved with interventions such as optimal or targeted medical therapy, aggressive preventive measures for risk factors, and early initiation of anti-ischemic medications [16].

Despite the high cardiovascular event rate in people with diabetes, not all of them have CAD. Systematic screening for myocardial ischemia cannot be recommended because of the high cost. Thus, the reason why a high-risk asymptomatic diabetic population should be defined is to fully benefit from screening [17]. Clinical diagnosis is poor because myocardial ischemia is asymptomatic in people with diabetes and resting ECG which is often used has a low sensitivity and specificity. It is not clear the most appropriate screening test for silent myocardial ischemia in diabetics as the different tests detect different stages of the ischemic cascade. However, stress ECG is recommended as a first-line test to screen for myocardial ischemia. When inconclusive, myocardial perfusion scintigraphy seems more appropriate [6]. A coronary angiogram should be performed in case of a positive stress test [6]. This is to guide further treatment modalities-percutaneous coronary angioplasty, coronary bypass surgery, or medical treatment alone. However, coronary angiography is very expensive and not available in our context. Stress electrocardiography is more available and affordable in our context and was considered for screening for silent myocardial ischemia in this study. Very little is known about the prevalence and determinants of silent myocardial ischemia in black Africans with type 2 diabetes. This study aimed to determine the prevalence and associated factors of silent myocardial ischemia in patients with type 2 diabetes in a resource-limited setting.

## Methods

This study is reported following the Standard for Reporting Observational Studies in Epidemiology (STROBE) checklist [18].

**Study design and setting:** between January and April 2019, we carried out a cross-sectional study at the National Obesity Centre (NOC) and the cardiology unit of the Yaounde Central Hospital (YCH). The YCH is one of the teaching hospitals of the Faculty of Medicine and Biomedical Sciences of the University of Yaounde I. The NOC is the largest endocrinology unit in the country. Patients were recruited at the National Obesity Centre (NOC) of the Yaoundé Central Hospital (YCH).

**Participants:** the participants were consenting patients with type 2 diabetes, of both sexes, aged ≥ 18 years who were receiving care for at least one year at the NOC. We excluded patients with myocardial infarction on resting ECG, history of coronaropathy, and terminal diseases. Those who could not do the exercise stress test due to rheumatologic diseases were also excluded.

**Procedure and measurements:** we carried out this work in three phases-Phase 1 (invitation and screening for eligibility), phase 2 (collection of clinical and biochemical data), and phase 3 (exercise stress ECG). The invitation was done during consultations of patients at the NOC. The potential participants were then screened for eligibility. Data were then collected and entered into a pre-designed and pre-tested questionnaire. Sociodemographic information (age, gender, occupation, ethnic group, the region of origin), clinical data on the history of diabetes, cardiovascular risk factors, medications, and blood pressure measurement were collected. Anthropometric data such as height, weight, waist, and hip circumference were collected. Laboratory results such as lipid profile, serum creatinine, and HbA1c were obtained from patients´ files. The eligible patients were then programmed to do the resting ECG and exercise stress testing on another day.

**Vital and anthropometric parameters:** blood pressure (mmHg) was measured after 5 minutes of rest, in the sitting position with the arm supported at the level of the heart, the arterial blood pressure was measured using an electric sphygmomanometer (OMRON® HEM-712 C, OMRON HEALTHCARE, INC. Bannockburn, Illinois 60015. CHINA). The mean of 2 values was recorded. Pulse (per minute) was manually taken at the left wrist on the radial artery with a stopwatch. Weight (kg) was measured with a SECA® balance scale in a standing position in light clothing and without shoes to the nearest 0.5kg. Height (m) was measured with a stadiometer in the erect position without shoes and socks. The average of two measurements was recorded. Body mass index (BMI) was calculated from the values of the height and the weight using Quetelet´s formula: Weight/Height^2^ and expressed in kg/m^2^ to the nearest one decimal place. Waist circumference was made in the standing position at the mid-distance between the lower costal margin and the anterior superior iliac crest. This was measured and expressed to the nearest 1cm. Body fat mass was measured by impedance meter using a calibrated fat monitor (OMRON BF 302, OMRON MATSUSAKA Co., Ltd Japan) to the nearest 0.1kg. The impedance meter has input variables: age, sex, height, and weight. Two measurements were carried out for percent body fat and body fat mass and in each case, the average value was used for data entry. Body surface area was calculated as (4w+7)/ (w+90), where w is the weight.

**Framingham risk score (FRS):** the Framingham score and CVD risk in percentage were calculated using the 2019 software Framingham risk score calculator. The following risk factors were considered to calculate the score: gender, age, total cholesterol, HDL cholesterol, LDL cholesterol, smoking history, and systolic blood pressure history of diabetes mellitus. The FRS was then used to calculate the cardiovascular disease (CVD) risk in 10 years. CAD risk was classified as high risk (CVD risk ≥ 20), intermediate-risk (CVD risk from 10 to 20), and low risk (CVD risk ˃ 10).

**Resting ECG and treadmill test:** a baseline 12-lead resting ECG was performed to assess for infarction. Only patients without ECG changes of myocardial infarction (pathologic Q waves in 2 concomitant leads) underwent the exercise stress test. This was performed on a treadmill using the Bruce protocol by an experienced cardiologist (LMF). Optimal conditions for the test were: No cigarette smoking 3 days before the test. No use of beta-blockers, nitrates, insulin, oral hypoglycemic agents, and no food before the test. A brief physical examination was performed to rule out any outflow obstruction and vascular bruit on the day of the test. Patients were allowed to run on the treadmill machine until at least the maximum heart rate for the patient was reached. The blood pressure was measured: Before starting the exercise as a baseline reference, once during each stage of the exercise, at the peak of exercise, at least twice in the recovery, or until blood pressure has returned to values similar to pre-exercise pressure. The stress test was stopped once the patient developed any of the following: Severe angina, sustained ventricular tachycardia, exertional hypotension, supraventricular tachycardia, severe hypertension (systolic BP ≥ 250 mmHg). The exercise test was considered as maximum if the patient reached 85% of the predicted maximum heart rate (MHR) according to the Gulati formula (Predicted MHR = 220-age). The test was considered positive if there was horizontal or down-sloping ST-segment depression of at least 1mm or 2mm upsloping ST depression that persisted for 0.06s to 0.08s after the J point, or if the patient developed angina symptoms like chest pain, dyspnea, or drop in systolic blood pressure ≥ 10mmHg. The test was considered non-diagnostic if the participant failed to reach at least 85% of the age-predicted maximum heart rate.

**Variables:** the main outcome variable was a positive exercise stress ECG test on a treadmill. This was diagnostic of silent myocardial ischemia. Silent myocardial ischemia is defined as the objective evidence of myocardial ischemia in the absence of chest discomfort or other angina equivalents. The potential determinants (predictive variables) of a positive test were the classical cardiovascular risk factors (clinical and biological), comorbidities, diabetes-specific characteristics (duration, treatment, level of control), and abnormalities on resting ECG [7,12,13,15].

**Sample size estimation:** we used the online calculator of sample size OpenEpi info version 3.0. Released April 4 and revised April 6, 2013. For a Hypothesized % frequency of outcomes in the population (P) =8% ± 5, a Confidence level (1 - α) =95%, and an 80% power to detect a significant association, the minimum sample need for this study was 90 patients with type 2 diabetes.

**Statistical analysis:** the data were analyzed using the statistical software IBM-SPSS version 20.0. Continuous variables are presented as means ± standard deviation (SD), or as median with interquartile range (IQR). Categorical data are presented as numbers and proportions. Chi-square and student T-test were used for statistical calculations for categorical variables. Multivariable analysis was used to look for factors independently associated with a positive stress test. Variables with a p-value < 0.2 in bivariate analysis were entered into the model. Statistical significance was considered as a p-value ≤ 0.05.

**Ethical statement:** ethical clearance was obtained from the Center Region Ethics Committee for Human Research (CRECH) (CE N° 751/CRERSHC /2019) and the Ethical Committee of the Faculty of Medicine and Biomedical Sciences, University of Yaounde 1 (Ref N° 154/UYI/FMSB/VDRC/CSD). Administrative authorization was obtained from the Director of the YCH. We carried out this work following the Declarations of Helsinki [19].

## Results

**Figure 1:**
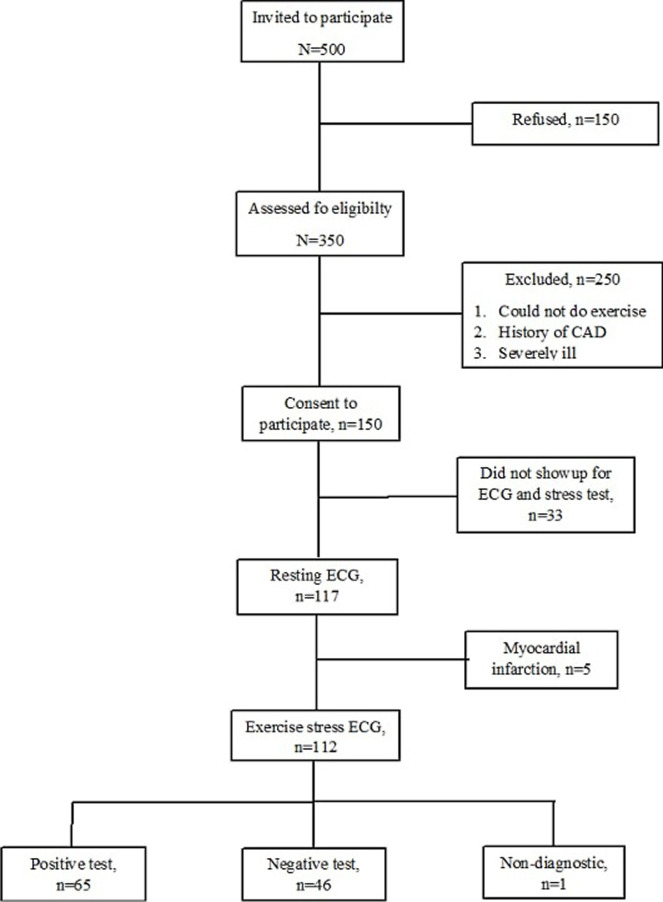
flow diagram of the participants

**Participants:** a total of 500 patients with type 2 diabetes were invited to participate in the study. Of these, 150 refused, 250 could not do physical activity, 5 had myocardial infarction on resting ECG, and 33 did not show up on the day of the stress test. 112 patients underwent exercise stress ECG. One patient failed to reach 85% of the age-predicted maximum heart rate (Figure 1).

**Sociodemographic and clinical characteristics:** a total of 112 patients with type 2 diabetes were retained for this study, with -63 (55.8%) of them being males. The median age was 58 (IQR: 51 - 64) years and range from 35 to 74 years. The median duration of diabetes was 8 (IQR: 5 - 12) years and ranged from 1 to 35 years. Dyslipidemia (69.0%) and a sedentary lifestyle (65.5%) were the most frequently reported cardiovascular risk factors (Table 1). Hypertension was reported by 50.4% of patients, and 70 (61.1%) patients had poor glycaemic control (HbA1c > 7%). Three or more cardiovascular risk factors were seen in 90 (80.3%) patients. Peripheral neuropathy was the most frequent complication of diabetes in 54% of patients. Only 1 (0.9%) patient was taking a beta-blocker. The median (IQR) HbA1c (%) was 8 (6.7 - 10), total cholesterol (g/l) was 1.63 (1.49 - 1.82), HDL cholesterol (g/l) was 0.44 (0.38 - 0.56), LDL cholesterol (g/l) was 1 (0.81 - 1.21), and triglyceride (g/l) was 1.33 (0.87 - 1.58). The 10-years median Framingham cardiovascular risk score was 10.0 (4.5 - 21.6). Fifty-one patients (45.9%) had low risk, 31 (27.9%) had intermediate-risk, and 29 (26.1%) had a high risk for CVD in 10 years. Left Ventricular Hypertrophy and T wave inversion were the most frequent anomalies on resting ECG (Table 1).

**Table 1:** cardiovascular risk factors, comorbidities, treatment, and baseline ECG characteristics of the study population

Variable	Frequency (n)	Percentage (%)
Other Cardiovascular risk factors		
Hypertension	57	50.4
Dyslipidemia	78	69.0
Tobacco use	23	20.4
Obesity	66	58.0
Abdominal obesity	50	44.6
Sedentary life>	74	65.5
Complications of Diabetes		
Retinopathy	18	15.9
Nephropathy	8	7.1
Peripheral neuropathy	61	54.0
Stroke	2	1.8
Diabetic foot	1	0.9
Treatment		
Statin	21	18.9
Aspirin	12	10.6
Beta blockers	1	0.9
Calcium channel blockers	28	24.8
ACE inhibitor	26	23.0
ARAII inhibitors	12	10.8
Thiazide diuretics	25	22.5
Oral antidiabetic agents only	73	64.6
Insulin only	12	10.6
Oral antidiabetic agents and insulin	27	23.9
Baseline resting ECG		
Left Bundle Branch Block	2	1.8
Left Anterior hemi-fascicular Block	5	4.4
Right Bundle Branch Block	6	5.3
Left Ventricular Hypertrophy	11	9.7
Left atrial enlargement	6	5.3
First degree AV block	4	3.5
T-wave inversion	11	9.7
Bradycardia	4	3.5
Tachycardia	1	0.9
Left axis deviation	8	7.1

**Prevalence of silent myocardial ischemia:** of the 112 patients who underwent the treadmill test, 65 (58%) had a positive stress test, 46 (41.1%) were negative, and 1 (0.9%) was non-diagnostic. Of those with a positive test, 61 had pathologic ST-segment depression, 1 had angina chest pain, 1 had a significant drop in blood pressure, and 2 had severe dyspnoea. The median (IQR) exercise time was 9 (8 - 10) minutes for a median (IQR) maximum speed of 2.7 (2 - 3.2) m/s. The median (IQR) age-predicted MHR% achieved was 92 (88-97)%, and the median (IQR) heart rate achieved was 151 (141-164) beats per minute. The median (IQR) METs achieved was 6.85 (5.5-8.0). The median (IQR) ST-segment depression was 1.4 (1 - 2.2) mV. The median (IQR) SBP was 160 (150 - 170) mmHg, and the DBP was 90 (85 - 95) mmHg. We found a significant association between the duration of diabetes of fewer than 10 years and myocardial ischemia (p-value = 0.03). A significant association was also found between female sex (p-value = 0.032), age < 60 years (p-value = 0.048) and silent myocardial ischemia by TMT (Table 2). There was no significant association between the complications of diabetes, poor glycaemic control, hypertension, dyslipidemia, tobacco use, obesity, abdominal obesity, sedentary lifestyle, and SMI by TMT (Table 3). In multivariable analysis, factors independently associated with a positive stress test were abdominal obesity (aOR: 4.2, [95% CI: 1.4 - 12.8]) and Female sex (aOR: 2.5, [95% CI: 1.1 - 5.7]) (Table 4).

**Table 2:** clinical factors associated with a positive treadmill test

Variables	Positive stress test, n (%)	OR (95% Confidence interval)	p-value
Sex			
Female	34 (70.8)	2.5 (1.1 - 5.6)	0.022
Male	31 (49.2)	1	
Age (years)			
<60 years	42 (66.7)	2.2 (1.01 - 4.7)	0.048
≥60 years	25 (52.1)	1	
Duration of Diabetes > 10 years			
Yes	19 (45.2)	0.4 (0.2 - 0.9)	0.026
No	46 (66.7)	1	
Risk factors ≥3			
Yes	49 (55.7)	1.15 (0.5 - 2.9)	0.763
No	12 (52.2)	1	
Framingham score ≥20			
Yes	16 (55.2)	0.8 (0.4 - 1.9)	0.667
No	49 (59.8)	1	
Body Mass Index ≥30kg/m^2^			
Yes	25 (67.6)	1.8 (0.8 - 4.1)	0.173
No	40 (54.1)	1	
Abdominal Obesity			
Yes	56 (62.9)	2.5 (0.6 - 2.8)	0.061
No	9 (40.9)	1	
Systolic Blood Pressure≥140mmHg			
Yes	17 (68)	1.7 (0.7 - 4.3)	0.276
No	48 (59.8)	1	
Diastolic Blood Pressure≥90mmHg			
Yes	18 (60)	1.1 (0.5 - 2.6)	0.851
No	47 (58)	1	
Pulse pressure ≥45mmHg			
Yes	30 (63.4)	1.5 (0.7 - 3.2)	0.281
No	35 (53.6)	1	
Pulse >70 beats/min			
Yes	48 (57.1)	0.78 (0.3 - 1.9)	0.593
No	17 (63)	1	
Neuropathy			
Yes	34 (55.7)	0.8 (0.4 - 1.7)	0.505
No	31 (62)	1	
Nephropathy			
Yes	6 (75)	2.2 (0.4 - 11.6)	0.327
No	59 (57.3)	1	
Retinopathy			
Yes	11 (61.1)	1.1 (0.4 - 3.2)	0.810
No	54 (58.1)	1	
Smoking			
Yes	53 (58.9)	0.9 (0.3 - 2.4)	0.823
No	12 (57.1)	1	
Sedentary life>			
Yes	35 (47.3)	0.4 (0.2 - 0.9)	0.022
No	26 (70.3)	1	
Alcohol use			
Yes	36 (52.9)	0.8 (0.4 - 1.8)	0.592
No	25 (58.1)	1	

**Table 3:** lipid profile and ECG pattern associated with a positive treadmill test

Variables	Positive stress test, n (%)	OR (95% Confidence interval)	p-value
Total Cholesterol >2g/L			
Yes	10 (66.7)	1.5 (0.5 - 4.7)	0.493
No	55 (57.3)	1	
LDLc >1g/L			
Yes	34 (61.8)	1.3 (0.6 - 2.8)	0.489
No	31 (55.4)	1	
HDLc <0.4g/L			
Yes	19 (55.9)	1.1 (0.5 - 2.4)	0.896
No	42 (54.6)	1	
Triglyceride >1.5g/L			
Yes	20 (57.1)	0.9 (0.4 - 2.1)	0.837
No	45 (59.2)	1	
Total cholesterol/HDLc ratio > 5			
Yes	9 (64.3)	1.3 (0.4 - 4.2)	0.641
No	56 (57.3)	1	
HbA1c>7%			
Yes	42 (60.9)	1.3	0.526
No	23 (54.8)	1	
Abnormal resting ECG			
Yes	41 (58.6)	1.5 (0.7 - 3.2)	0.317
No	20 (48.9)	1	

**Table 4:** factors independently associated with a positive treadmill test

Variables	aOR (95% Confidence interval	p-value
Abdominal obesity	4.2 (1.4 - 12.8)	0.011
Female sex	2.5 (1.1 - 5.7)	0.038
Age (for each 1 year)	1.04 (0.99 - 1.09)	0.115
Duration of diabetes (for each 1 year)	1.02 (0.95 - 1.1)	0.537

## Discussion

Diabetes is a major risk factor for developing coronary artery disease. Myocardial ischemia in people with diabetes is usually asymptomatic. The silent nature of this disease is due to cardiac autonomic neuropathy. Repeated episodes of silent ischemia lead to myocardial fibrosis, ventricular dysfunction, arrhythmias, and even sudden death. Early detection of silent myocardial ischemia (SMI) can therefore prevent these events [20]. This study aimed to determine the prevalence and associated factors of silent myocardial ischemia in type 2 diabetic patients in Cameroon, a low-income setting in sub-Saharan Africa. A total of 111 patients with type 2 diabetes followed up at the National Obesity Centre were recruited for this study. More than half of the patients had silent myocardial ischemia. We observed a significant association between age less than 60 years and silent myocardial ischemia. This result is contrary to that of Janand-Delenne *et al*. [21] and Yoo *et al*. [22] who found that increasing age was significantly associated with SMI by TMT. This could be explained by attrition bias which is unequal loss of participants due to dead or drops out of the study. A significant association was found between the female sex and myocardial ischemia. This finding is contrary to that of Rajagopalan *et al*. [23], Araz *et al*. [15] and De Lorenzo *et al*. [24] who showed that the male sex was a significant predictor of myocardial ischemia in T2DM. Lavekar *et al*. [13] did not find any significant association between gender and myocardial ischemia.

Duration of diabetes is an important risk factor for developing diabetes complications. Out of 69 participants with a duration of diabetes < 10 years, 46 (66.7%) had a positive stress test. We observed a significant association between the duration of diabetes < 10 years and the positive stress test. This result is contrary to that of Kim *et al*. [25] who found that CAD diagnosed by coronary angiography was higher in participants with a duration of diabetes > 10 years and elderly. Also, a positive association between the duration of diabetes and SMI has been reported by Langer *et al*. [26] and Sharda *et al*. [27]. The difference in results could be explained by the fact that there is no national screening program to diagnose diabetes, hence making it difficult to correctly determine the duration of diabetes.

The prevalence of silent myocardial ischemia in our study population was 58%. This is similar to that reported by Scognamiglio *et al*. [10] who found that the prevalence of asymptomatic coronary artery disease in T2D patients with greater than or equal to two CVD risk factors and less than or equal to one CVD risk factor was 59.4% and 60% respectively using dipyridamole myocardial contrast echocardiography (MCE). This is different from that obtained by the Milan study group with a prevalence of 12.1% [28]. In the largest study conducted to date, the DIAD study to assess the prevalence and predictors of silent myocardial ischemia in T2D patients by adenosine-stress single-photon emission-computed tomography (SPECT) myocardial perfusion imaging (rMPI) obtained a prevalence of 22% [7]. This difference could be explained by the difference in inclusion criteria. One of the main reasons for the discrepancy in prevalence might also be due to the different screening techniques used to detect myocardial ischemia in diabetic patients. These techniques have different sensitivity and specificities.

Although hypertension, obesity, and smoking, are well-known risk factors for CAD, we did not find any significant association between them and SMI. This observation is similar to that of the DIAD study to detect silent myocardial ischemia in asymptomatic diabetic patients who reported that there was no significant association between the traditional cardiovascular risk factors and SMI [7]. Our findings are also similar to that of Scognamiglio *et al*. [10]. Even though there was no significant association between CAD risk factors and myocardial ischemia, we observed that the prevalence of these risk factors in those with a positive stress test was higher than in those with a negative stress test.

Dyslipidemia is commonly seen in patients with type 2 diabetes. The prevalence of dyslipidemia in our patients was 69.1%. This was accentuated in those with positive TMT. Dyslipidemia, high total cholesterol level, high triglyceride level, high LDL, and low HDL, had no statistically significant association with myocardial ischemia. This is similar to the findings of Bacci *et al*. [29] and the DIAD study [7] which did not find any association between dyslipidemia and silent myocardial ischemia by TMT. On the contrary, the Milan study reported a significant association between serum triglyceride, serum cholesterol, and myocardial ischemia. Agarwal *et al*. [30] found that high serum LDLc had a significant association with SMI detected by TMT.

In the present study, there were more TMT cases among those subjects whose glycaemic control was not optimum as determined by HbA1c. More than half of our population (61.9%) had poor glycemic control (HbA1c ≥ 7%). However, there was no significant association between poor glycemic control and positive TMT. This finding is similar to that obtained by Bacci *et al*. [29] and the DIAD study [7] which did not report any significant association between poor glycemic control and silent myocardial ischemia. This suggests that though glycemic control may be important in preventing diabetes complications, it may not be an important contributing factor to myocardial ischemia. However, some contrasting results were obtained by Lavekar *et al*. [13] who showed that there was a significant association between poor glycemic control and silent myocardial ischemia.

Forty participants (64.5%) with normal resting ECG had positive TMT. However, there was no significant association between abnormalities on resting ECG and positive TMT. This result is similar to that reported by Sharda *et al*. [27] who also reported no significant association between abnormal ECG and myocardial ischemia. The MiSAD (Milan study on Atherosclerosis and diabetes) study showed a significant association between abnormal ECG at rest and myocardial ischemia [28]. Also, the American Diabetes Association recommends screening for CAD in T2D patients with abnormal resting ECG [31]. This difference could be because we considered abnormalities on resting ECG which are not directly associated with CAD.

This study has some limitations. This was a single-center cross-sectional study in a group of patients with type 2 diabetes. We cannot tell if the findings will be similar in other settings, characterized by ethnic diversity. The medium and long-term prognostic significance of a positive treadmill test is not known in our setting. There is a need for follow-up studies in this group of patients. The minimal sample size estimated was 90. This could still be small, thereby reducing the power to detect factors significantly associated with ST-segment depression on the treadmill. We also included patients with resting ECG abnormalities such as left bundle branch block and T-wave inversion. This could modify the ST-segment response with exertion. Due to inadequate finances, and limited technical plateau, it was not possible to do a complete evaluation of the patients to confirm ischemia. A family history of ischemic heart disease could not be obtained because of a lack of information or documented diagnosis. Also, it was not possible to determine peripheral artery disease with an ultrasound machine, cardiovascular autonomic neuropathy, and microalbuminuria. These are potential factors associated with ischemic heart disease. Despite these limitations, this study paves the way for further research on silent myocardial ischemia in patients with type 2 diabetes in a low-income setting.

## Conclusion

The prevalence of silent myocardial ischemia by treadmill test was high in this group of asymptomatic patients with a relatively short duration of type 2 diabetes. Factors independently associated with a positive stress test were abdominal obesity and female sex. The traditional cardiovascular risk factors were not associated with a positive stress test. The clinical implication is that all female diabetic patients with abdominal should undergo screening for silent myocardial ischemia. This strategy is however seems not cost-effective. There is also a risk of missing out other patients. Our study population might not be representative of the entire patients with diabetes in our setting. Thus, our findings cannot be generalized. This highlights the need for early diagnosis and management of patients with type 2 diabetes to prevent subclinical cardiovascular disease. Our findings should be confirmed in larger studies, using other affordable methods such as stress echocardiography coupled with stress ECG for screening silent myocardial ischemia.

### What is known about this topic

- Diabetes is a major public health problem worldwide with the greatest burden in low-income settings;
- Diabetes is associated with high cardiovascular morbidity and mortality;
- Patients with diabetes often present with silent myocardial ischemia with varying proportions and risk factors according to the setting.

### What this study adds

- One out of two patients with diabetes present with silent myocardial ischemia (positive treadmill test);
- The traditional cardiovascular risk factors were not associated with a positive test;
- Factors independently associated with a positive stress test were abdominal obesity and female sex.
